# Forcible Straightening of the Spine in Paraplegia

**Published:** 1902-12-27

**Authors:** 


### FORCIBLE STRAIGHTENING OF THE SPINE IN PARAPLEGIA.

A case is reported in the *British Medical Journal* by Dr. Outterson Wood and Mr. Cantlie, in which complete paraplegia was successfully treated by forcible straightening of the spinal column which was the seat of angular curvature. The patient was a child, aged ten years, who had been healthy until two years before his admission in November, 1900. In 1898 something was noticed wrong with his back, for which he was treated as an out-patient at a hospital and kept in a jacket for eighteen months, at the end of which time he was discharged cured. In March 1900 the boy's legs began to be stiff and he had difficulty in moving about, and in May of that year there was complete paralysis of both lower limbs. On admission in November, 1900, there was complete paralysis of both lower limbs, associated with marked rigidity and a good deal of adductor spasm, but there was no flexion of the knees or thighs. From the umbilicus downwards sensation was greatly diminished. Bladder and rectum functions were unaffected. There was an angular curvature in the region of the second, third, and fourth dorsal vertebræ, with a backward curvature of the cervico-dorsal region, as a whole. For three months the patient was kept on his back and extension was employed, massage being used to the affected limbs. After three months of this Mr. Cantlie applied forcible extension under anæsthesia. The boy was turned face downwards, and each of the upper and lower limbs was pulled upon by a nurse, Mr. Cantlie meanwhile applying his hand to the most prominent part of the curvature and exercising gradually increasing pressure until a sensation of something "giving way" was communicated to the hand. Then a plaster support was immediately applied and the patient was placed supine in bed with some degree of extension of the lower limbs. During the night, however, the patient complained so much of pain in the knee-joints that the extension was given up. In June the patient could move both legs, but could not lift them. Sensibility also was returning. Eight months after the operation there was still some angular curvature, although considerably less prominently in the upper dorsal region; but all signs of active disease had disappeared, and he could run and jump about and play with other children. There was also complete recovery as regards tactile and thermal sensations, and pain was correctly appreciated. The knee-jerks were still somewhat exaggerated, but there was no rigidity and no adductor spasm. In commenting upon this case Mr. Cantlie says that in performing the operation on cases of spinal curvature he makes it a rule to desist from pressure as soon as something is felt to have "given." Nothing more should be attempted; the complete reduction of the curvature, or the occurrence of a crack as if bony structures had snapped are not to be expected. The sensation experienced is as if some fibrous structure had been stretched and had given way. Were this rule observed, he says, there are but few instances in which, when paralysis is present, forcible extension may not be applied with safety and with every hope of good recovery.

